# Effects of a Commercial Microbial Agent on the Bacterial Communities in Shrimp Culture System

**DOI:** 10.3389/fmicb.2018.02430

**Published:** 2018-10-11

**Authors:** Zidan Liu, Linglin Qiuqian, Zhiyuan Yao, Xin Wang, Lei Huang, Jialai Zheng, Kai Wang, Laiguo Li, Demin Zhang

**Affiliations:** ^1^Key Laboratory of Applied Marine Biotechnology, Ministry of Education, Ningbo University, Ningbo, China; ^2^Medical School, Ningbo University, Ningbo, China; ^3^Chunlin Aquaculture Company, Ningbo, China

**Keywords:** shrimp, commercial microbial agent, planktonic bacterial communities, intestinal bacterial communities, high-throughput sequencing

## Abstract

Commercial microbial agents (e.g., probiotics, microbial products, microorganism preparation et al.) have been widely applied for disease control in shrimp culture. However, the effect of these microbial agents (MA) on shrimp health is unstable and the underlying mechanism remains unclear. The effect of MA can probably be achieved by influencing the bacterial community of shrimp culture system. To test this hypothesis, we used 16S rRNA gene amplicon sequencing to investigate the dynamics of both planktonic and intestinal bacterial composition in shrimp culture ponds with or without commercial MA applied weekly. The results showed that MA application increased the temporal turnover rate of bacterioplankton community. Within 1 week, MA-treatment significantly drove bacterioplankton community composition to divert from that without MA-treatment at day 2 after MA application, but the deviation tended to vanish at days 4 and 7. At day 21, a significant difference was observed in shrimp intestinal bacterial community between two groups. The relative abundance of Rhodobacteraceae in shrimp intestine was significantly greater in the MA-treated group than that in the control. However, MA-treatment did not significantly improve the growth or survival ratio of shrimp. This study suggest that MA works in terms of accelerating bacterioplankton community turnover and shifting intestinal bacterial community, however, its effect on shrimp growth might vary greatly and might be improved by optimizing the method in activation and application and more investigation on the microbial ecological process of shrimp culture system is needed before we develop and apply probiotics more efficiently.

## Introduction

Intensive shrimp culture is one of the dominant industries in coastal aquaculture of China. However, frequent occurrence of disease seriously limits its development (Balcazar et al., 2006). Chemicals, such as disinfectant and antibiotics, have commonly been used in intensive shrimp culture to control the prevalence of diseases, thus keeping high production (Ma et al., 2013). However, these strategies tended to result in antibiotic resistance of bacterial pathogens and potential risk of chemical residues in shrimps and often failed to result in high production (Gatesoupe, 1999; Cabello, 2006). Hence, it is crucial to establish environmentally friendly and practical technologies to control shrimp diseases and guarantee sustainable development of shrimp-culture industry.

Probiotics, defined as “live microorganisms which confer a health benefit on the host when administered in an adequate amount” (Newaj-Fyzul et al., 2014), have been widely used in human and veterinary medicine (Vine et al., 2006; Wang and Xu, 2006; Wang, 2007). The number of studies focusing on probiotics for aquatic animals is increasing with the demand for sustainable and environmentally friendly aquaculture (Gatesoupe, 1999). Some studies showed that probiotics could improve aquaculture water quality and maintain aquatic animals’ health through colonizing in their intestines (Ran et al., 2012; Silva et al., 2012). However, the conclusions on effects of microbial agents (MA) on shrimp-culture ecosystem differ from each other in various studies (Boonthai et al., 2011; Ma et al., 2013). Recently the application of molecular biology techniques such as DGGE and clone library showed significant advantages over the traditional culturing method in studies of microbial community composion (Liu et al., 2010, 2011; Chaiyapechara et al., 2012). However, they are still far from dealing with the huge amount of analytical flux of microbial diversity, and could reveal very limited information on microbial ecological mechanism of probiotics working.

Shrimps are in direct contact with planktonic microbes in surrounding water. Microorganisms play crucial roles in sustaining ecological balance (Zhang et al., 2014). Microbial growth cycle is short; thus, the community structure and function can change rapidly and sensitively with the slight change of the environment. In addition, microorganisms play a leading role in the material circulation, energy flow and the health maintenance of the whole culture system (Newaj-Fyzul et al., 2014). Some groups of bacterioplankton are important pathogens, such as *Vibrio, Rickettsiella*, and *Salmonella* (Cabello, 2006). A number of studies have indicated that some bacterioplankton showed a positive relationship with shrimp diseases and may serve as a biological indicator to evaluate the occurrence of shrimp diseases (Xiong et al., 2014a,b). Intestinal microbiota is also closely related to the health state of cultured aquatic organisms, either by food decomposition, antimicrobial compounds production and preventing of pathogen colonization (Boonthai et al., 2011; Chaiyapechara et al., 2012; Xiong et al., 2015). Moreover, bacterioplankton can strongly shape shrimp intestinal microbiota which is vital for shrimp nutrient, immunity, and disease resistance (Xiong et al., 2015). Therefore, variations in planktonic and intestinal bacterial communities could contribute to revealing the effect of probiotics and its underlying mechanism in improving shrimp health, thus providing scientific guidelines for the development and application of probiotics.

Although complex MA are frequently used in shrimp culture, there is little convincing evidence for how they work. In this study, we applied a popular commercial MA into multiple 3 m^3^ shrimp ponds with identical management through 21-day culture period to investigate how commercial MA works in shrimp culture and try to find evidences from: (i) the change of water chemical characteristics and the growth of shrimp and (ii) the dynamics of bacterioplankton community and shrimp intestinal bacterial community.

## Materials and Methods

### Experimental Design and Sample Collection

**FIGURE 1:**
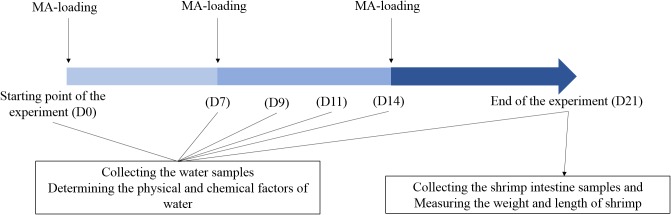
Experiment design across 21 days. D0, D7, D9, D11, D14, and D21 represent the days after initial application of MA.

Shrimp farm ponds investigated in this study are located in Zhanqi, Ningbo, China (29°32′N, 121°31′E). The test ponds are concrete and have the same size (1.6 × 1.8 × 2.0 m^3^ each) and uniformly managed in terms of sea water, daily water exchange rate (8%), water depth (1.2 m), shrimp rearing density (190 inds/m^3^), feed type (TECH-BANK, Yuyao, China) and schedule. The raw materials of the feed were mainly: fishmeal, soybean meal, peanut meal, wheat flour, squid paste, shrimp paste, shrimp shell powder, kelp powder, soybean phospholipid oil, fish oil, calcium dihydrogen phosphate, vitamin premix, mineral premix, etc. It has a crude protein content of about 40% and a crude fat content of about 3%. Shrimp (*Litopenaeus vannamei*) in middle size (length, 5.90 ± 0.14 cm; weight, 2.97 ± 0.75 g) were introduced into the ponds on 6 May, 2013. Ten ponds were randomly divided into two groups, the control group (CK) with a normal feeding mode and MA-treated group (Tre) with weekly application of a popular commercial MA (HUOJUNWANG, made by Hainan Zhuoyue Biotechnology company, China, which includes more than 10 species such as spore-forming bacteria, lactic acid bacteria, photosynthetic bacteria, actinomycete, etc). The MA was activated before application: 25 g MA and 25 g glucose (MA: glucose = 1: 1) were added to 5 L seawater (pre-disinfected by chlorine dioxide) and incubated for 12 h in the greenhouse. A little of activated MA fermentation broth (MA: feed = 1:100) was mixed into shrimp feed before applied to shrimp and the remaining added into pond water of the MA-treated group, 1 L for each pond. Later additional applications were weekly done. The daily management of this experiment has been illustrated in **Figure 1**.

The water samples were collected at six time points, corresponding to 0, 7, 9, 11, 14, and 21 days after shrimp inoculation. Approximately 1 L of water samples for DNA extraction was pre-filtered through nylon mesh (100 μm pore size). Subsequently, the samples were filtered onto a 0.2 μm polycarbonate membrane (Millipore, Boston, MA, United States) and the membrane was stored in -80°C until use. We first weighed all shrimps and then randomly picked up 30 individuals to determine the average length and weight. We calculate survival ratio of shrimps with the total weight and average individual weight. For intestine sampling, the shrimps were washed twice with alcohol, cut with a scalpel, and 10 intestines were picked out with sterile toothpick under aseptic condition, then mixed in a sterilized 1.5 mL EP tube. Samples were stored at 4°C and transported to the lab within 1 h, stored -80°C until use.

### Environmental Parameter Analyses

Temperature, Dissolved oxygen (DO) and pH were measured by a probe (YSI 550A, United States) in each pond every day. The concentrations of dissolved organic carbon (DOC), total dissolved phosphate (TDP), nitrite (NO_2_^-^), phosphate (PO_4_^3-^) and chemical oxygen demand (COD) were analyzed according to standard methods (GB 17378.4-2007).

### DNA Extraction

Bacterioplankton DNA was extracted using a Power Soil^®^ DNA isolation kit (MOBIO Laboratories, Carlsbad, CA, United States) and intestinal bacterial DNA by QIAamp DNA Stool mini kit (Qiagen, GmbH, Hilden, Germany). The DNA extraction of original MA and activated MA is the same as the intestinal bacterial DNA. The extracted DNA were quantified with NanoDrop ND-1000 spectrophotometer (NanoDrop Technologies, Wilmington, DE, United States) and stored at -80°C until amplification.

### Bacterial 16S rRNA Gene Amplification and Sequencing

An aliquot (50 ng) of DNA from each water sample was used as the template for amplification. The V3-V5 hyper variable regions of bacterial 16S rRNA gene (357F: CCTACGGGAGGCAGCAG and 926R: CCGTCAATTCMTTTRAGT) were amplified (Sim et al., 2012), the forward primer with 7 bp barcode sequences. Each sample was amplified in triplicate (in a 50 μL reaction system) under the following conditions: 30 cycles of denaturation at 94°C for 30 s, annealing at 55°C for 30 s and extension at 72°C for 30 s, with a final extension at 72°C for 10 min (Xiong et al., 2014a). PCR products of each sample were combined and purified with a PCR fragment purification kit (TaKaRa Biotech, Japan). An equimolar amount of PCR products for each sample were combined in a single tube and run on a Roche FLX 454 pyrosequencing machine (Roche Diagnostics Corporation, Branford, CT, United States), producing reads from the forward direction 27F with the barcode.

For shrimp intestinal and MA samples, the V4 hyper variable regions of bacterial 16S rRNA gene (515F: 5′-GTGTGCCAGCMGCCGCGG TAA-3′ and 806R: 5′-GGACTACHVGGTWTCTAT-3′) that include the Illumina flow cell adapter sequences were amplified (Minseok et al., 2011), the forward and reverse amplification primer with 6 bp barcode sequence. The PCR amplication and DNA purification is the same as described in the last paragraph. The sequencing was done using the MiSeq sequencing platform (Illumina, San Diego, CA, United States).

### Processing of Sequence Data

All the sequences generated in this study were deposited in the Sequence Read Archive of NCBI^1^ and are available under accession number (SRR3944126). For 454 sequencing samples, sequencing reads were quality filtered and chimera checked using the Quantitative Insights into Microbial Ecology (QIIME v1.7.0) workflow (Caporaso et al., 2010). Specifically, the bacterial reads whose length was outside the bounds of 200 and 450 bp and cases in which the homopolymer run exceeds 6 were removed by “split_libraries.py” (Reeder and Knight, 2010), then sequences with the same barcode were assigned into the same sample (Caporaso et al., 2010). Bacterial phylotypes were identified using uclust (Edgar, 2010) and assigned to operational taxonomic units (OTUs, 97% cutoff). The most abundant sequence from each phylotype was selected as the representative sequence and was aligned using PyNAST (DeSantis et al., 2006). Taxonomic identity of each phylotype was determined using the Greengenes database (DeSantis et al., 2006). To avoid the deviation caused by various sequencing depth, we randomly selected a subset of 6100 sequences per sample for downstream analyses. Across the water samples, we obtained a total of 627,516 high-quality sequences and 6,100–21,700 sequences per sample (mean = 10,458; *n* = 60).

For MiSeq sequencing, data quality was controlled using the PyroNoise (DeSantis et al., 2006) script with default settings. Using Greengenes database as reference, the remaining sequences were chimera detected and removed using Usearch (Edgar et al., 2011). Bacterial phylotypes were assigned to operational taxonomic units (OTUs; 97% sequence similarity) using the “pick_de_novo_otus.py” script with Uclust method (Edgar, 2010). The most abundant sequence of each phylotype was selected as the representative sequence and then was taxonomically assigned against Greengenes database (DeSantis et al., 2006). The “filter_taxa_from_otu_table.py” script was used to discard archaea, chloroplast, and singleton OTUs. We used a randomly extracted subset of 24200 sequences per sample for downstream analyses. Across the intestinal samples, we obtained a total of 599,481 high-quality sequences and 24,200–92,300 sequences per sample (mean = 59,948; *n* = 10).

### Statistical Analysis

QIIME was used to calculate the diversity indices, including observed species, Shannon index and phylogenetic diversity. Pielou’s evenness was computed with PRIMER v5 (PRIMER-E Ltd, Plymouth, United Kingdom). Two-way analysis of variance (ANOVA) was applied to investigate the effect of MA application, culture time and their interaction with environmental variables and alpha-diversity indices using SPSS 16.0. The principal coordinates analysis (PCoA) based on Bray-Curtis distance was applied to evaluate the difference in bacterioplankton community structure and shrimp intestinal bacterial community structure between two groups using Past. Permutational multivariate analysis of variance (with “adonis” function) based on Bray-Curtis distance was performed to partition the community variance constrained by culture time, MA-treatment and their interaction using ‘vegan’ package in R (Statistical Package 2009; R Development Core Team, 2010). The time-decay model was used to evaluate the temporal turnover rate of bacterioplankton community composition with the power law model *S* = *cT^w^* (Green et al., 2004). Among the parameters, *w* is considered as an index of temporal turnover rate of bacterioplankton community and it is estimated with a linear regression based on the function expressed in log-log scale: log10S = log10*c* + *w*log10*T* (S represents similarity of the bacterioplankton community based on Bray-Curtis distance, T stands for the interval between two sampling time). Mixed model analyses of covariance (ANCOVA) was applied to test the influence of MA-treatment on the turnover rate of bacterioplankton community composition using SPSS 16.0. Using the “labdsv” package in R^2^, screening the OTUs with the largest indicating value on top 10 which make significant differences between the two groups (*P* < 0.05). The significantly discriminant taxa of the intestinal bacterial communities in each treatment were determined using the least discriminant analysis (LDA) and discriminant taxa were used to generate taxonomic cladograms illustrating differences between treatments.

## Results

### The Effect of Activation on MA Components

In original MA product,the main families were Bacillaceae, Lactic aic-bacteria (Lactobacillaceae, Leuconostocaceae, Enterococaceae and Streptococcaceae), and Rhodobacteraceae (**Supplementary Figure S1**). After activation, Streptococcaceae is the only family whose relative abundance significantly increased (2.1% up to 13.1%) among the main families of original MA and reached the second larger family in the activated MA culture. In the contrary, Bacillaceae, Lactobacillaceae and Enterococaceae decreased significantly (28.2–3.9, 10.9–0.5, 3–0.03%, respectively), and apparent drops also happened with the relative abundance of Leuconostocaceae and Rhodobacteraceae (11.3–5.7, 7.7–3.9%, respectively) (**Supplementary Figure S1**). These resulted in a total reduction of main original MA bacteria from 73.7 to 27.8% in the activated MA culture. The resulting vacancy was filled mainly by Vibrionaceae (37.4%), which became the top abundant family in the activated MA culture. In addition, Ectothiorhodospiraceae (4.5%) and Bradyrhizobiaceae (3.0%) was also newly recruited.

### Chemical Characteristics of Water Samples and the Growth of Shrimp

Overall, chemical parameters of the water samples were highly temporally variable in the culture ponds (**Supplementary Figure S2**). NO_2_^-^, PO_4_^3-^, DTP, and COD increased, while DOC fluctuated during the experiment. The temperature of the culture ponds is maintained at 23–26°Ñ. However, the MA application showed no significant effects on these variables.

The survival ratio of shrimp and feed conversion ratio (FCR) showed no significant difference between the control and MA-treated group (*P* = 0.485 and *P* = 0.195, respectively, **Table 1**). At the 21st day, the average length and weight of shrimp in both groups also showed no difference between two groups (**Table 1**).

**Table 1:** Survival ratio, feed conversion ratio, length and weight of shrimps after 21 days.

	Tre	CK	*P* (*t*-test)
Survival ratio (%)	66.0 ± 8.2	62.4 ± 7.0	0.485
FCR	1.6 ± 0.2	1.8 ± 0.2	0.195
Length (cm)	8.47 ± 0.14	8.48 ± 0.11	0.903
Weight (g)	7.57 ± 0.12	7.58 ± 0.10	0.890


*The data represents the mean ± standard deviation (n = 5); Survival ratio equals the number of survived shrimp divided by initial number (survived shrimp number was calculated by total weight and individual weight), FCR, feed conversion ratio of shrimps. CK represents control group, Tre represents MA-treated group.*

### Taxa Distribution and Alpha Diversity of Bacterial Community

**FIGURE 2:**
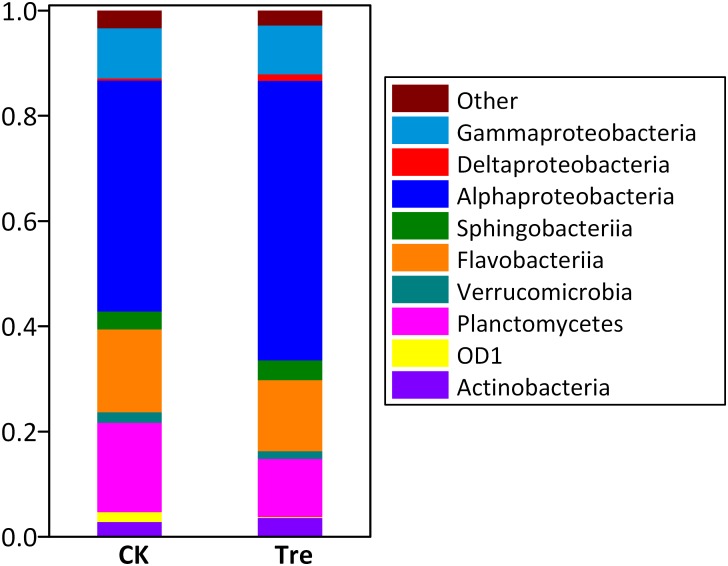
Average relative abundances of the dominant bacterial phyla/classes (Proteobacteria and Bacteroidetes are assigned to the class) (relative abundance > 1%) in intestinal samples (*n* = 5) at day 21. “Others” represent the phyla with less than 1% of relative abundance. CK represents control group, Tre represents MA-treated group.

Across the water samples, the dominant (relative abundance > 1% at least in one sample) phyla/classes of bacterioplankton community were Alphaproteobacteria, Sphingobacteriia, Flavobacteria, Deltaproteobacteria, Gammaproteobacteria, and Actinobacteria (**Supplementary Figure S3**). Two-way ANOVA showed that the relative abundances of all the dominant phyla significantly changed during the experimental duration. MA application significantly influenced the relative abundance of Actinobacteria (*P* = 0.016) and Gammaproteobacteria (*P* = 0.005). As to shrimp intestinal samples, the dominant phyla/classes were Alphaproteobacteria, Bacteroidetes, Gammaproteobacteria and Planctomycetes (**Figure 2**). Among these, the relative abundances of Deltaproteobacteria (*P* = 0.007) and Planctomycetes (*P* = 0.010) were significantly influenced by the application of MA.

Two-way ANOVA showed that the alpha-diversity of bacterioplankton communities was significantly influenced by culture time and MA-treatment (*P* < 0.05; **Supplementary Table S1**). All the alpha-diversity indices showed a sharp increase from days 0 to 7 (**Supplementary Figure S4**). In general, the alpha-diversity of the MA-treated group was lower than the control, except day 9. For shrimp intestinal samples on day 21, the alpha-diversity was not significantly influenced by MA-treatment (*P* > 0.05, *t*-test).

### Bacterial Community Structure

**FIGURE 3:**
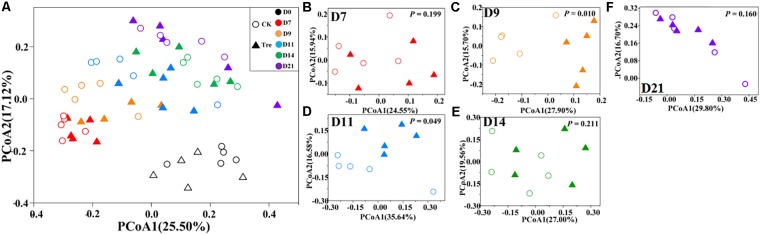
Principal coordinates analysis (PCoA) plots based on Bray-Curtis distance across different sampling days. **(A)** Presents all water samples; **(B–F)** stands for D7, D9, D11, D14, and D21, respectively. CK represents control group, Tre represents MA-treated group. The *P*-value in **(B–F)** indicates differences of community structure between two groups. *P* < 0.05 indicates significant difference in community structure between two groups.

**FIGURE 4:**
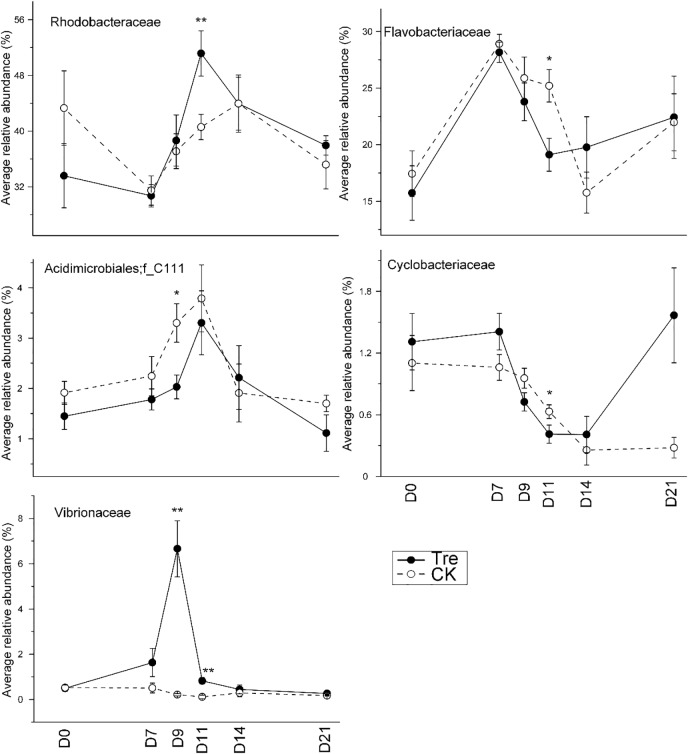
Average relative abundance of the dominant families (relative abundance > 1%) in water samples over the sampling days. CK represents control group, Tre represents MA-treated group. At each sampling time point, the relative abundance of each family requires a *t*-test between two groups. ^∗^*P* < 0.05, ^∗∗^*P* < 0.001.

**FIGURE 5:**
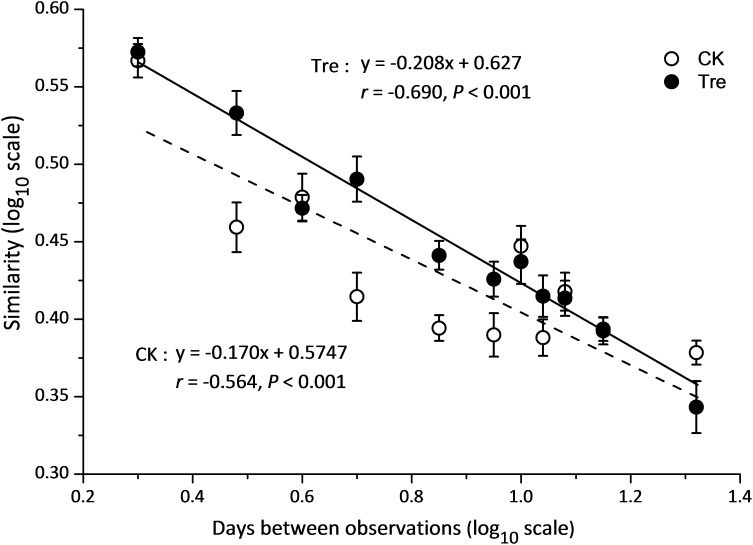
Similarity-time decay models of bacterioplankton communities. The power-law exponent *w* was estimated directly with a linear regression (log-log space approach) fit between the average Sørensen similarity values and intervals of sampling time. CK represents control group, Tre represents MA-treated group. The mixed model ANCOVA showed that MA-treatment accelerated the temporal turnover of bacterioplankton community compositions as compared to the control (*P* < 0.05).

Across the water samples, the bacterial community structure was more dissimilar among sampling times than between groups (**Supplementary Table S2**), which was corroborated by a dissimilarity test (perMANOVA). The culture time explained 16.2% of the community variation (*P* < 0.05), while MA-treatment and their interaction explained 2.3 and 1.6%, respectively. In addition, the short-term effect of MA-treatment on bacterioplankton community was evaluated in the second week. In the middle of the second week (days 9 and 11), dissimilarity in the bacterial community between two groups was clearly observed but tended to be less obvious afterward (**Figure 3A**). This pattern was further confirmed by ANOSIM, which showed that the bacterial community of MA-treated ponds was significantly different (*P* < 0.05) from the control on days 9, 11, but converge on day 14 (**Figures 3B–E**). Furthermore, the relative abundances of dominant families (relative abundance > 1% in at least one group) also significantly varied between two groups in the middle of second week (days 9 and 11) (**Figure 4**). The relative abundance of Rhodobacteraceae and Vibrionaceae were significantly higher in the MA-treated group, while it was lower for Flavobacteriaceae, Acidimicrobiales and Cyclobacteriaceae (**Figure 4**). The temporal successions of bacterioplankton community compositions fitted to time-decay model with the turnover rate of 0.170 and 0.208, for the control and MA-treated group, respectively (**Figure 5**). The mixed model ANCOVA showed that MA-treatment accelerated the temporal turnover of bacterioplankton community compared to that in the control (*P* < 0.05, **Figure 5**).

**FIGURE 6:**
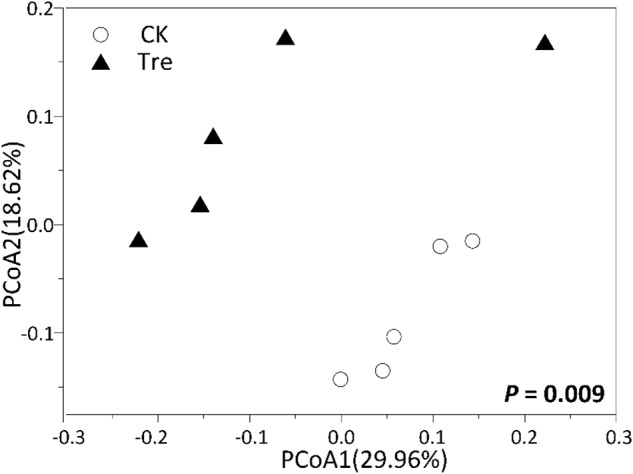
Principal coordinates analysis (PCoA) plots based on Bray-Curtis distance on the 21st day (*n* = 5). CK represents control group, Tre represents MA-treated group. The *P*-value indicates differences of community structure between the two groups. *P* < 0.05 indicates significant differences in community structure between two groups.

**FIGURE 7:**
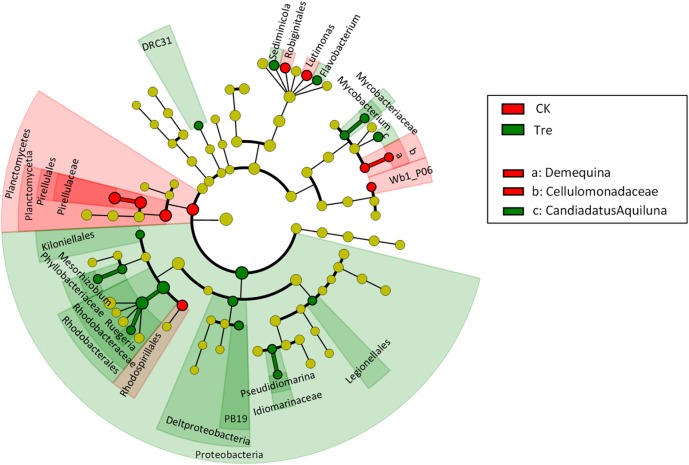
Least discriminant analysis (LDA) effect size taxonomic cladogram comparing all intestinal samples categorized by two groups. Significantly discriminant taxon nodes are colored and branch areas are shaded according to the highest ranked group for that taxon. If the taxon is not significantly differentially represented among sample groups, the corresponding node is colored yellow. CK represents control group, Tre represents MA-treated group.

For shrimp intestinal samples on day 21, the PCoA plot revealed pronounced differences in bacterial composition between two groups, primarily separated by the first axis (**Figure 6**). Similarly, ANOSIM also showed a significant difference between two groups (*P* = 0.009). Furthermore, we identified the key OTUs for characterizing MA-treatment (**Supplementary Table S3**). In MA-treated group, the indicative OTUs mainly belonged to Rhodobacteraceae while OTUs of the CK was more dispersely distributed in taxa (**Supplementary Table S3**). Certain taxa belonging to Alphaproteobacteria such as Phyllobacteriaceae and Rhodobacteraceae were enriched in MA-treated group, whereas others geared to Planctomycetes - Pirellulaceae showed lower abundance in MA-treated group compared to the control (**Figure 7**).

## Discussion

### Activation Method Significantly Affects the Bacterial Community Composition of MA Fermentation Broth

HUOJUNWANG, a popular MA used in many Chinese shrimp farms, was selected for the investigation of the effect of probiotics on the bacterial community succession in shrimp culture system. In order to increase the amount of effective probiotic bacteria, activation is the regular step before its application into feed and pond water. Here, we activated HUOJUNWANG according to the manufacturers instructions. Strikingly, the relative abundance of most expected effective bacteria decreases greatly and Vibrionaceae instead bloomed to be the most dominant taxa in the activated MA culture (**Supplementary Figure S1**). Vibrios are normally related with disease occurrence in aquaculture (Ken et al., 2009; Zhang et al., 2014). Although there was no apparent disease happened in this experiment, the activation method here may counteract the beneficial effect of HUOJUNWANG. Studies have also shown that the effect of MA on water quality varied with different activation methods (Wang et al., 2009). Therefore, more attention should be paid on MA activation method in future. The better activation method might be developed based on the nutrient requirements and appropriate culturing condition of target probiotics. On the other hand, the more species mixed in MA, the more difficult to screen one universal method. It might be better to separate the probiotic bacterial species into subgroups and activate them separately. Anyway, activation method is one of critical steps for MA working in shrimp farm.

### The Effect of MA on Water Quality and Shrimp Growth Indices

The effect of probiotics is an important topic. Probiotics is used widely in aquaculture but, compared with livestock breeding, the evaluation of probiotics effect and efficiency is more difficult due to the infeasibility to real-time get the growth parameters (such as total weight, survival ratio, growth rate et. al). So there are often controversy reports about the effect of MA on water quality and aquaculture animal growth. Some studies have shown that probiotics could improve the water quality and provide good surroundings for shrimp growth (Chaiyapechara et al., 2012; Melgar Valdes et al., 2013; Newaj-Fyzul et al., 2014). For instance, Wang et al. (2005) demonstrated that *Bacillus* sp., acted as a protein ammonifying bacterium, significantly reduced the dissolved active-phosphorus, total inorganic nitrogen and chemical oxygen demand in aquaculture water. However, Nimrat et al. (2012) showed that *Bacillus* application did not significantly improve the water quality of shrimp-culture system. Shariff et al. (2001) noted that the use of *Bacillus* sp. did not significantly improve the survival ratio of *Penaeus monodon*, while Ziaei-Nejad et al. (2006) showed that the *Bacillus* spp. increased the survival ratio of shrimp. In the present study, we did not observe significant improvement on water quality and shrimp growth indices by MA-treatment. Generally, the water exchange rate was relatively low with the studies in which MA showed positive effect. For instance, Melgar Valdes et al. (2013) changed water once a week (5–10%), while Wang et al. (2015) didn’t change water during the whole experiment. However, for the negative result, the water exchange rate was quite high, similar to our result (Nimrat et al., 2012; Zheng et al., 2017). Hence, the non-effectiveness of MA might attribute to the frequent water exchange and the short culture time, leading to low accumulation of eutrophication stress which might be not strong enough for MA-treatment effect to manifest. Another explanation might be MA activation method. During MA activation the unexpected Vibrionaceae grew much faster than target effective Bacillaceae (**Supplementary Figure S1**) which plays an important role in improving water quality. Accordingly we did not observe higher relative abundance of Bacillaceae in MA-treated pond water. Instead Vibrionaceae propagated much more in MA-treated group (**Figure 3**) which might counteract the role of Bacillaceae.

These results might largely arise from the unsuccessful colonization of probiotics in water and/or the shrimp intestine (Xiong et al., 2016). More studies in the dynamics of planktonic and intestinal bacterial communities in shrimp culture system with MA application will give scientific evidences for more effective application for MA.

### The Effect of MA on Bacterioplankton Community Composition Was Short-Lived

High temporal variation in the composition of bacterioplankton community was observed in this study but did not conceal MA effect (**Figure 3** and **Supplementary Table S2**). However, MA-treatment resulted in significant change of bacterioplankton community structure in a short time and then the divergence gradually converges (**Figure 3**). This may be due to the resilience of bacterioplankton community to the external disturbance which has been widely reported (Shade et al., 2012; Chen et al., 2016). As to the shrimp-culture system, the applied MA was exogenous microorganism, acting as external disturbance. The microbial community presents a certain degree of recovery when the disturbance disappears or the disturbance intensity tends to decrease. As a result, the effect of MA on bacterioplankton structure is not permanent. We suspect that if the relative abundances of MA applied into ponds are greater by improving MA activation method, the effect of MA-application could be stronger but still short-lived on bacterioplankton community. Hence, it is critical to figure out the right application frequency to ensure persistent effect in the future work.

### MA Shifted the Intestinal Bacterial Community

Shrimp intestinal microbial community plays a pivotal role in conditioning intestinal micro-ecological balance (Clemente et al., 2012; Newaj-Fyzul et al., 2014) due to its high relevance with the capability of digestion and absorption of nutrients, regulation of immune response and eventually maintenance of shrimp health (Thompson et al., 2010; Wang and Gu, 2010). The effect of MA-treatment on intestinal bacterial community was more durable compared with the bacterioplankton community (**Figures 3A, 6**). Rhodobacteraceae showed higher abundance in MA-treated group compared with the control (**Supplementary Table S3** and **Figure 7**), which is consistent with the changes in the bacterioplankton community (**Figure 4**). Rungrassamee et al. (2014) showed that host’s intestine has a stronger selection on intestinal bacteria than the bacterioplankton community in the culturing environment. Hence, the significant difference of intestinal bacterial communities between two groups might be a comprehensive reflection of the changes of bacterioplankton community. The shrimp intestinal tract receives water and food that are populated with microorganisms from the surrounding environment, demonstrated that shrimp intestinal bacterial community was undoubtedly affected by the resident microbiota (Wang et al., 2014). Rhodobacteraceae plays an important role in water purification and reducing harmful substances of water (Nupur et al., 2013). Rhodobacteraceae, as a specific taxon of MA-treated group in shrimp intestine, is associated with algae, and some members of Rhodobacteraceae can produce tropodithietic acid (TDA) to inhibit the growth of pathogen (Beyersmann et al., 2017). In addition, the poor colonization of probiotics in shrimp intestine could also matters. Future investigation should focus on improving colonization of probiotics and whether the probiotics works in shrimp-culture systems with high stress.

## Conclusion

In this study, we tried to validate the role of MA in shrimp culture from planktonic and intestinal bacterial communities. The results showed MA-treatment significantly affected both of the bacterioplankton community and intestinal bacterial community. The impact of MA on bacterioplankton community was short, while its effect remained longer for the intestinal bacterial community. However, no significant positive results of MA-treatment were observed on shrimp growth performance and water quality. MA effect may vary greatly with the activation method and application frequency of MA, the density and total biomass of cultured shrimp, and water pollution stress, etc. Systematic and in-depth research in the dynamics of planktonic and intestinal bacterial communities in shrimp culture system with MA application will give scientific evidences for more effective application of MA.

## Author Contributions

DZ and XW provided the experimental ideas and design of this study. LQ, LH, ZL, JZ, and LL did the experiments. LQ and ZL collected the experimental data and performed the laboratory analyses. ZL, ZY, KW, and LQ wrote the manuscript. DZ revised the manuscript.

## Conflict of Interest Statement

The authors declare that the research was conducted in the absence of any commercial or financial relationships that could be construed as a potential conflict of interest.

## Footnotes

1 http://www.ncbi.nlm.nih.gov

2 http://www.r-project.org
