# Supplementary material for: Effects of a Commercial Microbial Agent on the Bacterial Communities in Shrimp Culture System
Source: Front Microbiol. 2018 Oct 11;9:2430. doi: 10.3389/fmicb.2018.02430 (PMC6193131; doi:10.3389/fmicb.2018.02430)
Supplement: Supplementary file 1 [file Presentation_1.PDF]

Supplemental Data Summary

Table S1 Two-way analysis of variance (ANOVA) for alpha-diversity indices of bacterioplankton communities across different sampling days and MA-loading [a].

		Observed species	Shannon index	Pielou's evenness	Phylogenetic diversity
Time	F	8.515	8.318	7.866	6.871
	P	**< 0.001**	**< 0.001**	**< 0.001**	**< 0.001**
MA-loading	F	4.271	8.122	8.097	6.432
	P	**0.044**	**0.006**	**0.007**	**0.015**
Time × *MA-loading*	F	0.915	0.283	0.735	0.748
	P	0.479	0.920	0.601	0.592

[a] The data represents the mean ± standard deviation across water samples. Bold values represent significant difference of bacterioplankton community diversity between two groups at the level of Time, MA-loading and Time × MA-loading ($P < 0.05$).

Table S2 Permutation multivariate analysis of variance (with ADONIS function) based on Bray-Curtis distance between communities.

	Time		Treatment		Time × Treatment	
	R^2	*P*	R^2	*P*	R^2	*P*
Community variance	0.162	**0.001**	0.023	0.088	0.016	0.286

[a] R^2 values represent the proportion of variance constrained by factors. Bold values represent significant difference of bacterioplankton community structure between two groups ($P < 0.05$)

Table S3 The detailed information of indicator taxa (OTUs) of the shrimp intestinal microbial community [a].

OTU NO.	Taxa	Group	Indval	*P*
OTU 56390	Planctomycetes; Planctomycetia; Pirellulales; Pirellulaceae; g_Unclassified	CK	1	0.013
OTU 57398	Actinobacteria; Acidimicrobiia; Acidimicrobiales; C111; g_Unclassified	CK	0.9	0.037
OTU 27229	Alphaproteobacteria; Rhodobacterales; Rhodobacteraceae; g_Unclassified	CK	0.8	0.044
OTU 54160	Bacteroidetes; Flavobacteriia; Flavobacteriales; Flavobacteriaceae; g_*Muricauda*	CK	0.8	0.044
OTU 14999	Planctomycetes; Planctomycetia; Planctomycetales; Planctomycetaceae; g_*Planctomyces*	CK	0.8	0.044
OTU 98089	Alphaproteobacteria; Rhodobacterales; Rhodobacteraceae; g_Unclassified	CK	0.8	0.043
OTU 106998	Bacteroidetes; Flavobacteriia; Flavobacteriales; Flavobacteriaceae; g_Unclassified	CK	0.8	0.034
OTU 33394	Bacteroidetes; Flavobacteriia; Flavobacteriales; Flavobacteriaceae; g_Unclassified	CK	0.8	0.027
OTU 105090	Actinobacteria; Actinobacteria; Actinomycetales; Cellulomonadaceae; g_*Demequina*	CK	0.8	0.021
OTU 100398	Bacteroidetes; Flavobacteriia; Flavobacteriales; Flavobacteriaceae; g_Unclassified	CK	0.8	0.013
OTU 30244	Alphaproteobacteria; Rhodobacterales; Rhodobacteraceae; g_Unclassified	Tre	1	0.006
OTU 65593	Alphaproteobacteria; Rhodobacterales; Rhodobacteraceae; g_Unclassified	Tre	1	0.022
OTU 78316	Alphaproteobacteria; Rhodobacterales; Rhodobacteraceae; g_Unclassified	Tre	0.9	0.005
OTU 50155	Alphaproteobacteria; Rhodobacterales; Rhodobacteraceae; g_Unclassified	Tre	0.9	0.011
OTU 88478	Alphaproteobacteria; Rhodobacterales; Rhodobacteraceae; g_Unclassified	Tre	0.9	0.016
OTU 56627	Alphaproteobacteria; Rhodobacterales; Rhodobacteraceae; g_Unclassified	Tre	0.9	0.022
OTU 19508	Actinobacteria; Actinobacteria; Actinomycetales; f_Unclassified	Tre	0.9	0.022
OTU 103088	Alphaproteobacteria; Rhodobacterales; Rhodobacteraceae; g_Unclassified	Tre	0.9	0.024
OTU 72110	Alphaproteobacteria; Rhodobacterales; Rhodobacteraceae; g_Unclassified	Tre	0.9	0.026
OTU 30561	Alphaproteobacteria; Rhodobacterales; Rhodobacteraceae; g_Unclassified	Tre	0.8	0.017

[a] CK represents control group; Tre represents MA-treated group; Indval stands for the influence by taxa on shrimp intestinal microbial community.

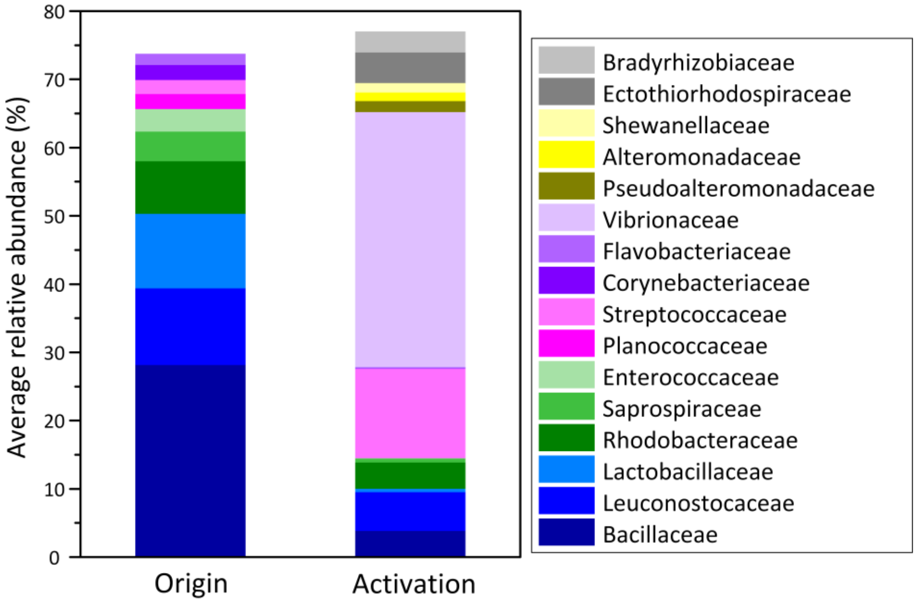

Fig. S1 Relative abundances of the dominant bacterial families (top 10) in MA before and after activation (Origin, before activation; Activation, after activation).

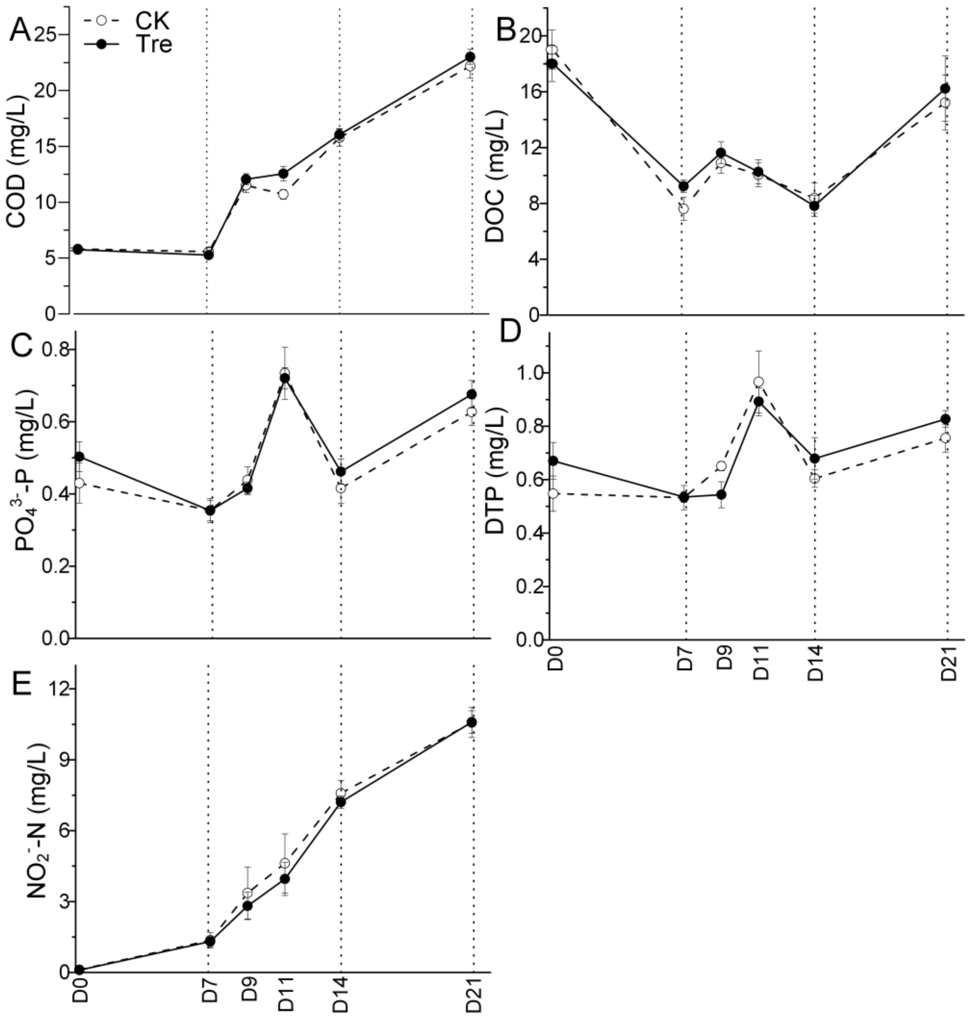

Fig. S2 Dynamics of nutrients over sampling days. MA-loading has no significant influence on all of the parameters (*t*-test, all *P* >0.05). COD: chemical oxygen demand;.DOC: dissolved organic carbon; DTP: total dissolved phosphate; CK represents control group; Tre represents MA-treated group. The data represent the mean ± standard deviation (n = 5).

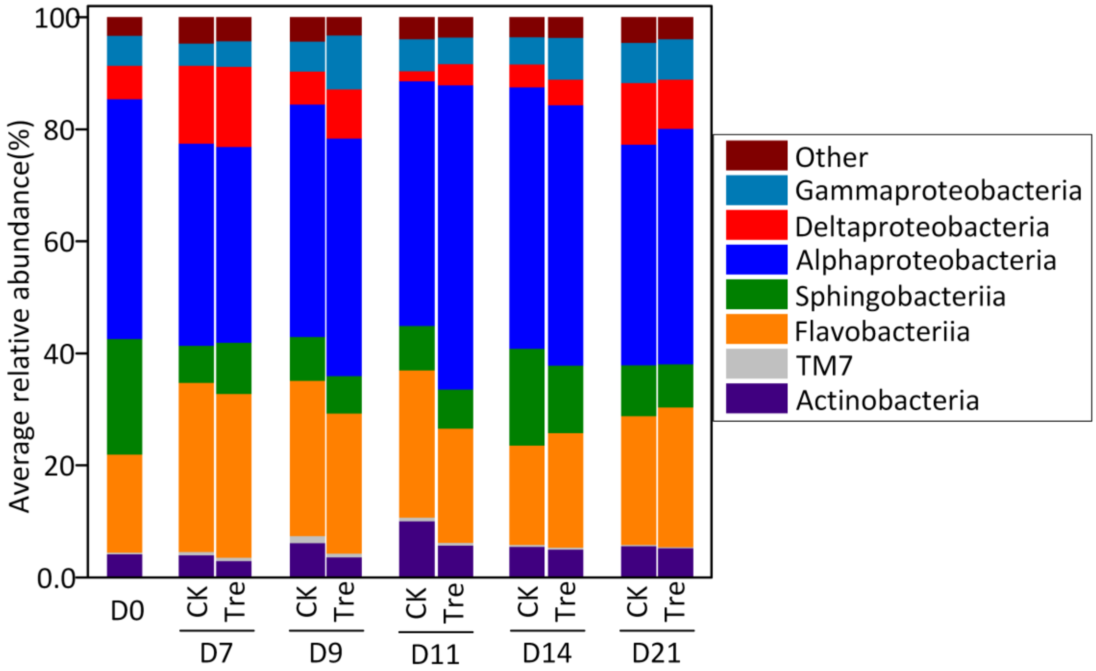

Fig. S3 Average relative abundances of the dominant bacterial phyla/classes (Proteobacteria and Bacteroidetes are assigned to the class) (relative abundance > 1%) in water samples. 'Others' represent the phyla with less than 1% of relative abundance. CK represents control group, Tre represents MA-treated group.

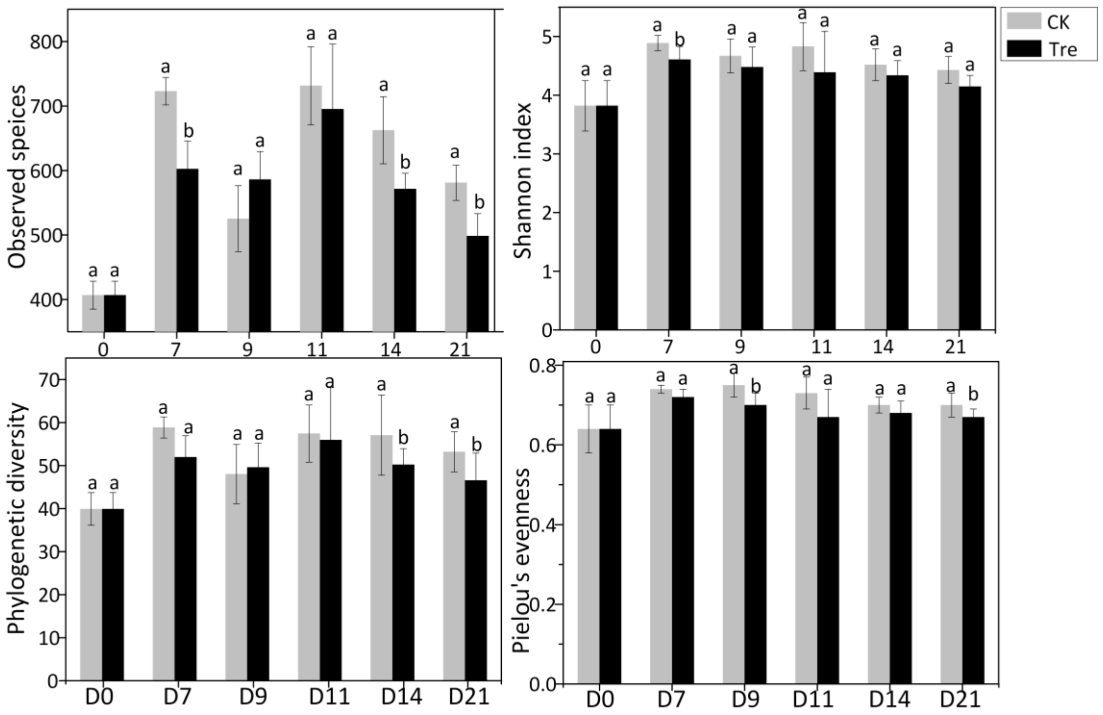

Fig. S4 The alpha-diversity indices of bacterioplankton communities across different sampling days and MA-loading. The data represent the mean ± standard deviation across water samples (n = 5). Different lowercase letters in the same sampling day indicate significant differences at 0.05 level. CK represents control group, Tre represents MA-treated group.
